# Self-Help for the General Practitioner

**Published:** 1919-02-01

**Authors:** 


### The Hospital

The Workers' Newspaper of Administrative Medicine and Institutional Life, Administration, National Insurance and Health.

No. 1704, Vol. LXV. SATURDAY, FEBRUARY 1, 1919. PRICE TWOPENCE

### SELF-HELP FOR THE GENERAL PRACTITIONER.

The position of the general or family medical practitioner in relation both to other departments of the professional hierarchy and to his patients and the public has been much canvassed in recent years, and prophecies and proposals on this topic have been numerous and at times mutually contradictory. On the one hand the practitioner has been told that his function is largely exhausted, and that the future lies not with generalism, but with specialism. On the other is the assurance that a larger equipment of knowledge and skill and wider opportunities of usefulness are bound to be attached to general practice, and that to press for these is the true policy. If the former of these views is accepted there is an end of the matter, and no discussion relative to improvement or advance is worth while. But for those who, like ourselves, are strongly convinced that medicine as a function in the life of the community demands a large body of well-equipped general practitioners, such an attitude of despair is impossible. It is of the public we think rather than of the doctors, though it must, of course, be remembered that unless the rewards of medical practice—and we refer not merely to financial rewards—are kept at a sufficiently high level, worthy and capable men and women will not elect for the service. In some degree the measure or quality of these rewards does rest with the public, and sooner or later the community, by fixing these, will have the doctors they deserve. There is, therefore, more than a professional interest in recent discussions, and there are signs that certain leaders of public opinion are beginning to recognise this. Yet when all is said and done that can be said and done from the outside, there still remains the question whether the general practitioners themselves are doing whatever is in their power to meet the situation. The gods, we are told, help those who help themselves, and the maxim may fairly be applied to the present discussion. Complaints are numerous. We would fain see more active effort and enterprise.

On the financial side of the debate we have, for the present, nothing to say. It will never be so attractive as to determine by itself the choice of the best minds. There must be added to it the prospect of a sufficient status and the certainty of increasing opportunities for self-realisation and for interesting and serviceable work. Without these, general practice will be too dull, too tiresome, and too unremunerative to draw to its service other than disciples of the second- or third-rate order. There is just now a general complaint that these opportunities are diminishing rather than increasing. The patients are gathered into special hospitals where the general practitioner may not follow them, or they are attracted to so-called "specialists." Further, the complexities of diagnosis and treatment are increasing, and the practitioner has in corresponding measure a difficulty in keeping himself abreast of the movements of the day and in equipping himself in the newer methods. He is becoming, so it is said, a mere finger-post whose only function is to direct patients to which hospital or "specialist" they should carry the burden of their woes. If there is exaggeration in all this, there is also some measure of truth. The attractiveness of general practice is without doubt diminishing, and, as we believe it is a public interest that it should be increased, we venture to suggest to practitioners on their own behalf, as well as on behalf of the public, that they should put their shoulders to the wheel. In the last resort the issue will be settled by a capacity to meet the needs of the public, and unless this condition is satisfied protests and aspirations will alike be in vain.

Leaving on one side for the moment the general question of organisation for the purpose of maintaining personal and professional interests against unfair political or corporate pressure, there are, it seems to us, two ends which general practitioners should seek to realise. The first of these is increased opportunities for what is commonly called postgraduate study. Most of such practitioners are reasonably near a hospital where more or less systematic clinical and pathological work is daily in progress. They ought to be so related to the hospital to be able to benefit from the work conducted within its walls, instead of, as at present, regarding themselves as unwelcome intruders. Here lies the opportunity for a man of determination to equip himself with knowledge and skill in almost any department of practice, and such opportunity, steadily cultivated, will soon fit him for dealing with the enormous majority of the cases which may come under observation. He may not boast, say, the dexterity to extract a cataract, but in the recognition and treatment of the ordinary diseases of the eye he will speak with authority. And what is true of ophthalmology is equally true of other branches. The public is continually needing help of this order, and the interest of the practitioner is to fit himself to supply such help. If he has repeatedly to avow his incompetence he must expect to be regarded as fit to deal only with minor ailments. But it is said, and with some truth, that even when he has acquired some special training the practitioner does not get a sufficient number of special cases to keep his skill at an expert level. It is here that general practitioners have a second chance to show themselves alive to the needs of the time. By partnerships or some looser union they can arrange among themselves to help one another in this respect, and can thus retain a considerable quantity of work which now slips out of their hands. So long as they remain isolated and mutually distrustful they must appeal, not to one another, but to some outside authority. The chance is for each to cultivate some particular branch of work in addition to a general capacity, and for a number in union to work together for a common advantage.

